# Automatic detection of foreign body objects in neurosurgery using a deep learning approach on intraoperative ultrasound images: From animal models to first in-human testing

**DOI:** 10.3389/fsurg.2022.1040066

**Published:** 2022-11-30

**Authors:** Haley G. Abramson, Eli J. Curry, Griffin Mess, Rasika Thombre, Kelley M. Kempski-Leadingham, Shivang Mistry, Subhiksha Somanathan, Laura Roy, Nancy Abu-Bonsrah, George Coles, Joshua C. Doloff, Henry Brem, Nicholas Theodore, Judy Huang, Amir Manbachi

**Affiliations:** ^1^ Department of Biomedical Engineering, Johns Hopkins University School of Medicine, Baltimore, MD, United States; ^2^ Department of Neurosurgery, Johns Hopkins University School of Medicine, Baltimore, MD, United States; ^3^ Department of Biomedical Engineering, Johns Hopkins University, Baltimore, MD, United States; ^4^ Department of Engineering Science, University of Toronto, Toronto, ON, Canada; ^5^ Roy Illustration, Des Moines, IA, United States; ^6^ Applied Physics Lab, Johns Hopkins University, Laurel, MD, United States; ^7^ Department of Materials Science and Engineering, Johns Hopkins University, Baltimore, MD, United States; ^8^ Department of Electrical Engineering and Computer Science, Johns Hopkins University, Baltimore, MD, United States; ^9^ Department of Mechanical Engineering, Johns Hopkins University, Baltimore, MD, United States; ^10^ Department of Anesthesiology, Johns Hopkins University School of Medicine, Baltimore, MD, United States

**Keywords:** deep learning, neurosurgery, ultrasound, foreign body object detection, computer vision

## Abstract

Objects accidentally left behind in the brain following neurosurgical procedures may lead to life-threatening health complications and invasive reoperation. One of the most commonly retained surgical items is the cotton ball, which absorbs blood to clear the surgeon’s field of view yet in the process becomes visually indistinguishable from the brain parenchyma. However, using ultrasound imaging, the different acoustic properties of cotton and brain tissue result in two discernible materials. In this study, we created a fully automated foreign body object tracking algorithm that integrates into the clinical workflow to detect and localize retained cotton balls in the brain. This deep learning algorithm uses a custom convolutional neural network and achieves 99% accuracy, sensitivity, and specificity, and surpasses other comparable algorithms. Furthermore, the trained algorithm was implemented into web and smartphone applications with the ability to detect one cotton ball in an uploaded ultrasound image in under half of a second. This study also highlights the first use of a foreign body object detection algorithm using real in-human datasets, showing its ability to prevent accidental foreign body retention in a translational setting.

## Introduction

Leaving behind surgical items in the body is considered a “never event” (1), yet it markedly burdens both patients and hospitals with millions of dollars spent every year on medical procedures and legal fees, costing $60,000 to $5 million per case (2–4). Nearly 14 million neurosurgical procedures occur annually worldwide (5), and in each craniotomy surgeons may use hundreds of sponges or cotton balls to clear their field of view. Thus, it is unsurprising that surgical sponges are the most commonly retained items (6). Unfortunately, retained foreign body objects may lead to life-threatening immunologic responses, require reoperation, or cause intracranial textilomas and gossypibomas, which mimic tumors immunologically and radiologically (7–10). Locating cotton balls on or around the brain becomes increasingly challenging as they absorb blood, rendering them visually indistinguishable from the surrounding tissue. Unlike larger gauze pads, which are often counted using radiofrequency tagged strips (11), cotton balls are small (closer to 10 mm in diameter), must be counted manually by nurses in the operating room as they are placed in and extracted from the open wound, and may leave behind a small torn strip of cotton. There is therefore an unmet need for an intraoperative, automatic foreign body object detection solution that can be streamlined into the neurosurgical workflow. Due to their prevalence in surgical procedures and the difficulties associated with tracking their use, cotton balls serve as an excellent model of retained foreign bodies inside the cranial cavity.

Although *seeing* the contrast between blood-soaked cotton balls and brain tissue poses a challenge, they can be distinguished by *listening* to them. Prior work has demonstrated that ultrasound is able to capture the different acoustic characteristics between these materials and interpret them via filtering and logarithmic compression to display distinctly on an ultrasound image (12). More specifically, ultrasound captures the difference in the acoustic impedance between brain parenchyma and cotton as a result of their distinct densities and the speed at which sound travels through them (acoustic impedance is the product of material density and speed of sound). Ultrasound is non-invasive, non-radiating, clinically available, inexpensive, portable, and able to display images in real time. Therefore, ultrasound is an optimal modality for visualizing and localizing retained cotton during neurosurgery.

Deep learning (DL) has shown promise in object localization within an image (13); therefore, a DL algorithm using ultrasound images holds exceptional potential as a solution to fill this clinical need. However, medical images have notably high resolution, complexity, and variability as a result of alternative patient positions and respiration artifacts. In general, ultrasound is widely considered as a relatively difficult imaging modality to read, as specialized sonographers must undergo years of training for certification to operate clinical machines, and the same goes for trained radiologists to read these images. Hence, DL with medical ultrasound images (e.g., diagnosis, object detection, *etc.*) can be particularly complicated and computationally expensive. A previous DL approach to cotton ball detection used a model called YOLOv4 (14) and reported an accuracy of 60% (15).

**Figure 1:**
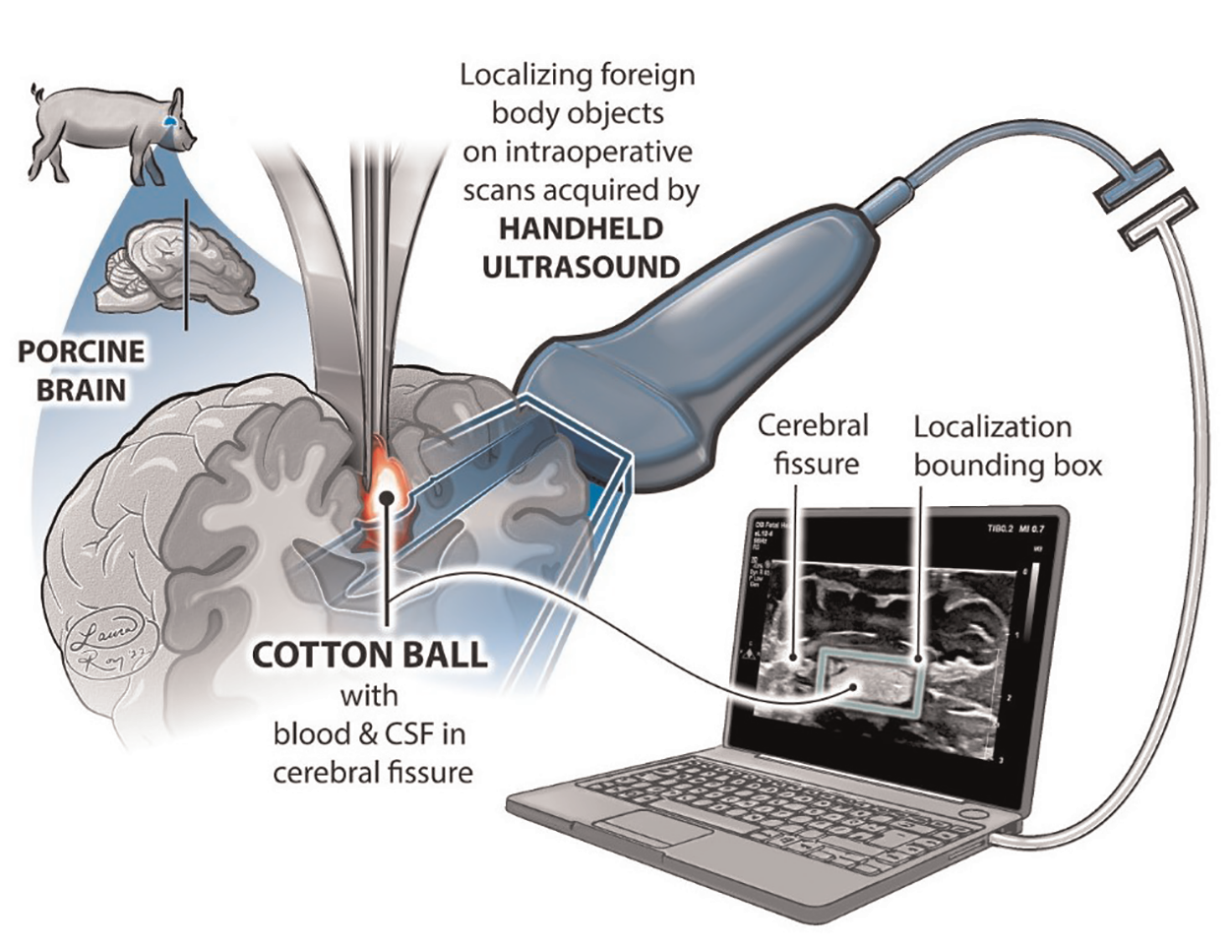
Foreign body object detection in neurosurgery. This study demonstrates the ability of medical ultrasound to locate foreign bodies, specifically cotton balls, retained in the brain post-surgery. Source: Laura Roy, CMI.

Here we present a highly accurate (99%), rapid (<0.5 s) ultrasound-based technology for both detection and localization of retained cotton in brain tissue (see summary Figure 1). We demonstrate the necessity of its inclusion in clinic via human studies, as a cotton ball not initially visible to a neurosurgeon in the surgical site was clearly observed in ultrasound images and subsequently removed from the cavity. This algorithm was able to identify that cotton ball. Ultrasound images acquired using a clinical ultrasound machine may be loaded into a web application hosted on a local server or captured with a smartphone camera using a custom app, which both reach a trained deep neural network that performs nonlinear regression and outputs the same image with one bounding box enclosing a cotton ball (if one exists). By localizing retained surgical objects within an ultrasound image, this method can distinguish between small fragments of cotton and folds in the brain parenchyma. It also could bring ease during long and intensive surgeries by alerting a clinician who may not be trained in sonography to a particular region of interest in an image, thus acting as an assistive device. First ever in-human studies show that this algorithm is already clinically relevant and ready to be incorporated seamlessly into neurosurgeries, with broad implications in medicine. This study paves the path for improved patient outcomes, minimal surgical errors, and reduction of the need for revisionary procedures and associated healthcare costs.

## Materials and methods

### Data acquisition

#### Animal study setup

**Figure 2:**
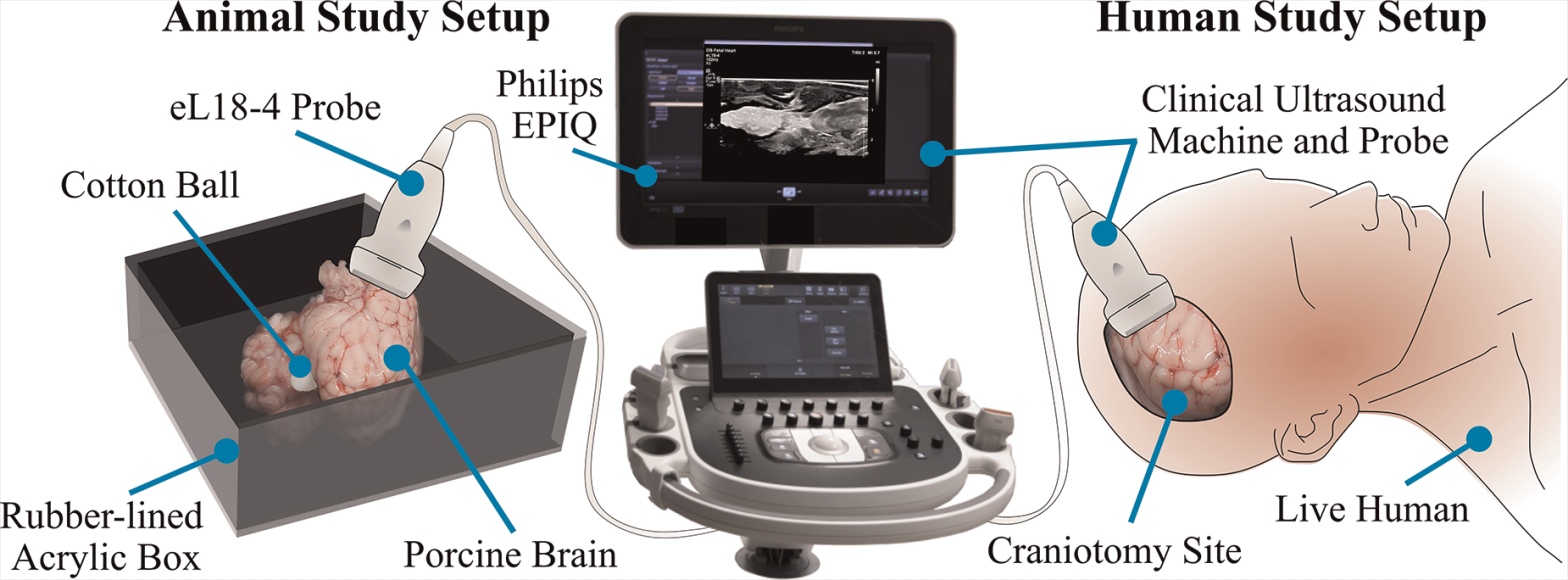
Experimental setup. Both animal studies and in-human studies were conducted. Animal images were acquired by setting an *ex vivo* porcine brain in a rubber-lined acrylic box, placing cotton balls within the brain, and immersing in saline. An eL18-4 probe of a Philips EPIQ 7 ultrasound machine was used to capture these images (left-hand side). The human studies were performed during neurosurgical procedures using an Aloka Alpha 7 machine and UST-9120 probe. The cranial cavity was scanned for retained cotton balls (right-hand side).

The algorithm used throughout this study was developed and tested using *ex vivo* porcine brain images. Porcine brains (Wagner Meats, Maryland, USA) were obtained and imaged with implanted cotton balls within 24 h of euthanasia. Prompt post-mortem imaging was necessary to avoid transformations in the acoustic properties of the brain tissue (16), which would change how the ultrasound machine interprets the image. These brain samples (N=10) were placed in a rubber-lined acrylic container filled with 1X pH 7.4 phosphate-buffered saline (PBS, ThermoFisher) to minimize artifacts in the recorded images. For imaging, an eL18-4 probe was used with a Philips EPIQ 7 (Philips, Amsterdam, Netherlands) clinical ultrasound machine (Figure 2).

Different sizes and locations of the cotton balls were imaged to mimic a neurosurgical procedure, including a control group with no cotton balls. Cotton balls were trimmed to diameters of 1, 2, 3, 5, 10, 15, and 20 mm. Approximately the same number of still images were captured for each size of cotton ball, with more control images to stress the importance of recognizing true negatives (i.e., understanding when there is not a cotton ball in the image). One saline-soaked cotton ball was implanted in the porcine brain per true positive image. To improve the variability among the images, the cotton ball was implanted at depths between 0 mm (placed directly underneath the transducer, above the brain) to approximately 40 mm (placed at the bottom of the container, beneath the brain). During imaging, the probe was moved and rotated around the outer surface of the brain to provide additional variability in the location of the cotton ball in the ultrasound image.

Additionally, experiments ensured that the acoustic properties of cotton in an *ex vivo* setting were representative of an *in vivo* setting, i.e., when soaked in blood during neurosurgery. Ultrasound imaging compared a 20 mm diameter cotton ball soaked in PBS with one soaked in Doppler fluid (CIRS, Norfolk, VA, USA, Model 769DF). Doppler fluid is designed to mimic the acoustic properties of blood. These images were compared visually by eye and by average pixel intensity value of the cotton ball, which would help ensure the DL algorithm could recognize cotton retained in an *in vivo* setting. The acoustic properties of cotton, PBS, and Doppler fluid were also assessed to confirm that the images should look similar based on the equation for acoustic impedance, which is used by the ultrasound machine to translate sound waves into image pixels.

Finally, other materials were tested using the technology developed here as well. A latex glove fragment (5 mm diameter), a stainless steel rod (5 mm diameter and 18 mm length), and an Eppendorf tube (7 mm in diameter and 30 mm in length) were placed on or around a porcine brain, imaged using ultrasound, and tested using the same methods as the cotton balls.

#### *In vivo* human studies

Ultrasound images of live human brains (N=2) were captured prior to closing the cranial cavity following (1) an aneurysm surgery and (2) a glioblastoma tumor resection. These images were acquired as part of a standard protocol by the neurosurgeon. Images were de-identified prior to being provided for evaluation of cotton ball presence, and this evaluation was conducted post-operatively (i.e., not as a part of the surgery).

The ultrasound machine available to the operating rooms was the Aloka Prosound Alpha 7 with a UST-9120 probe (Hitachi Aloka Medical America, Inc., Wallingford, CT). For the purposes of this study, a 10 mm diameter cotton ball was momentarily placed in the location of suturing or tumor removal, and saline was used to eliminate potential air bubbles prior to capturing the ultrasound images, which proceeded as follows. First, the neurosurgeon tasked with acquiring the ultrasound images identified the region of interest, i.e., the surgical site where a foreign body was known to have been placed. In a general case without a known cotton ball placement, this region of interest would be the open cranial cavity. The ultrasound probe was placed at the start of this window, with the depth adjusted to avoid image artifacts due to skull bone (Figure 2). During these surgeries, cine (moving) image scans were captured and later saved slice-wise as still images, although still images may be acquired as these are the input to this study’s custom algorithm. The probe was tilted in increments of 15${}^{\circ }$, taking care again to avoid bone reflections. To ensure that the entire region of interest is checked for cotton ball presence, the neurosurgeon translated the probe across the contour of the anatomy. The neurosurgeon slid the probe in 1cm increments in both the anterior-posterior and lateral directions until the entire exposed surface of the brain was covered. Once the cotton ball intentionally placed in the cavity was removed, a follow-up ultrasound scan captured another set of true negative images. During the second patient’s procedure, another cotton ball was visible on the ultrasound images. Thus, that cotton ball was found and removed in order to acquire images absent of any foreign body objects. Later, the de-identified images annotated by clinicians were checked for presence of a cotton ball using the developed web application.

### Data preprocessing

All images were annotated with ground truth bounding boxes surrounding cotton within the brain by researchers who conducted the study. The ground truth porcine brain images served as data for the DL model, split randomly but evenly by cotton ball diameter into 70% training set, 15% validation set, and 15% test set. Images were processed using anisotropic diffusion, which emphasizes edges while blurring regions of consistent pixel intensity, scaled from (768, 1024, 3) to (192, 256, 3) to decrease the computational power required to process each image, and normalized to pixel intensity values between 0 and 1. Each pixel in an image has an associated red, green, and blue color value, thus lending to the 3-dimensionality of the image. Intraoperative neurosurgical images in humans captured with a lower resolution probe additionally underwent contrast-limited adaptive histogram equalization (CLAHE) with a clip limit of 2.0 and a tile grid of side length 4 to increase image contrast.

### Algorithmic design

To ensure DL was in fact the optimal method for localizing cotton balls within ultrasound images, multiple less computationally expensive methods were implemented for comparison. These included thresholding and template matching. Because cotton appears brighter than most brain tissue in ultrasound images, an initial threshold at half the maximum of all grayscale pixel values was attempted (17). Additionally, Otsu thresholding was implemented as a method for identifying a natural threshold in the image (18). Finally, the average pixel values within ground truth bounding boxes of the training set images were calculated, and the images in the test set were thresholded at the 95th percentile of these averages. To implement template matching, four examples of different cotton balls were cropped from training set images to serve as “templates.” These template images were moved across each image of the test set at various scales from 25% to 200% the size of the template, and the location with the highest correlation value (most similar pixels) was taken to be the location of the cotton ball in the test set image (19). As an additional method for comparison, CSPDarknet53 (20), the DL backbone of YOLOv4 used in Mahapatra et al. (15), was implemented.

Ultimately, a fully automated DL algorithm for object localization was developed and packaged in a web application. DL is implemented in the form of neural networks, which are series of differentiable functions called “layers” that transform an input into a desired output. Convolutional neural networks (CNNs) are tailored towards image analysis. A CNN known as VGG16 (21) has shown success at reducing large medical image files to a few meaningful numbers. Here, a custom version of this model was used to predict four numbers from each ultrasound image representing: (1) the x value of the top left corner of the annotated bounding box, (2) the y value of the top left corner of the bounding box, (3) the width of the bounding box, and (4) the height of the bounding box.

**Figure 3:**
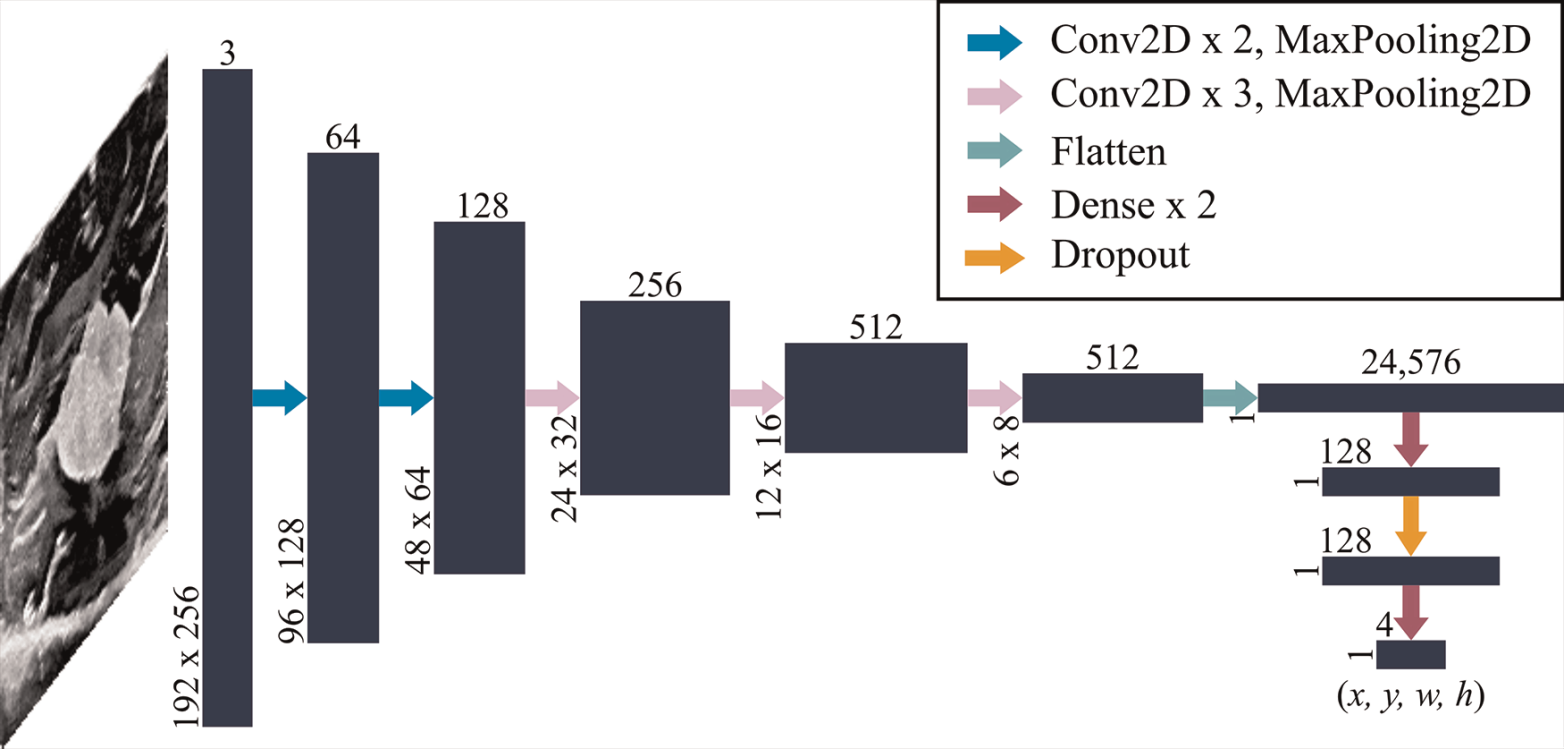
Network architecture. A convolutional neural network known as VGG16 was used as a backbone with an additional dense network split with a layer of dropout. Vertically and horizontally displayed number values indicate the 3-dimensional sizes of each layer (navy blue). The input is a 192×256×3 array (where the depth of 3 represents red, green and blue values assigned to each pixel) that is transformed to an output that is 1×4. These 4 values represent the *x* and *y* coordinates of the upper left corner of the predicted bounding box as well as the width and height of the box. Each arrow represents a function applied to the preceding layer as indicated in the legend. Convolutional layers were implemented with a 3×3 kernel size; max pooling layers used a 2×2 stride size; and dropout was 50%.

**Figure 4:**
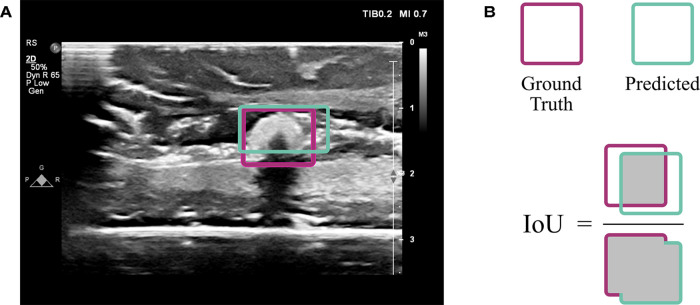
Evaluation using intersection over union (IoU). (**A**) An ultrasound image from the test set indicates the overlap of ground truth annotation of the cotton ball (magenta) with the predicted result by the CNN (sea green). (**B**) The extent of overlap is assessed by computing the area of overlap divided by area of union, known as the IoU.

The VGG16 model was customized by fine-tuning it and appending additional layers. When fine-tuning, pre-trained weights are used throughout most of the model except for the final few layers. These weights tell the network what to look for in an image. In a typical neural network, the initial layers tend to look more broadly at curves and lines, while the latter layers are trained to recognize high-level features specific to the task at hand, such as textures and shapes. By learning new weights for the last few layers, the network is able to be applied to new tasks; this process is known as fine-tuning (22). Thus, the network designed here implemented VGG16 using ImageNet weights (which are included in the Keras DL package (23)) for all layers except the last four, which remained “unfrozen,” or trainable. Additionally, five layers were appended to the VGG16 network: four dense layers split by a dropout layer (Figure 3). All convolutional layers and the first three dense layers used ReLU activation (22), while the final dense layer used sigmoid activation (22). The dropout layer was implemented with 50% dropout. Unlike convolutional layers, which learn local patterns, dense layers are fully connected and therefore learn global patterns in the data. Dropout layers help to regularize the model, i.e., improve its ability to generalize across images beyond the training set (22). A data generator shuffled the training set as it prepared batches of 64 images for each training step. The network passed each of the training set images through its layers 50 times, where each pass through the set is known as an epoch. Each of the 50 epochs had $\left\lfloor n_{imgs}/64\right\rfloor$ steps. An Adam optimizer (24) was implemented with a learning rate of 0.001. This parameter indicates the amount by which the weights in the model can change after each epoch, where a smaller learning rate also implies that the network learns more slowly. The loss function used to train and optimize the network was mean squared error. Results were evaluated using the intersection over union (IoU), which is the ratio of the overlap in predicted and ground truth bounding boxes to the combined total area of these boxes (Figure 4). An accurate prediction was considered one with an IoU over 50% (25). In addition to running the custom network on the randomly assigned training, validation, and test sets described above, stratified 5-fold cross validation (CV) was implemented to avoid overfitting. This method divided the entire set of images collected into five groups with randomly but evenly distributed cotton ball sizes. Each group took a turn as the test set, with the other four serving together as the training set. Mean IoU, accuracy, sensitivity, and specificity were calculated for each of the five models trained and averaged to get a final, cross-validated result. CV was performed on each of the compared neural networks. Gradio (26), a Python (RRID:SCR_008394) package, was used to develop an intuitive web-based interface that fits into the clinical workflow. The smartphone application was designed using Flutter, powered by Dart (27). All training was performed using a NVIDIA RTX 3090 GPU using Keras (23) and Tensorflow (28) (RRID:SCR_016345).

## Results

In addition to highly accurate cotton ball detection in *ex vivo* porcine brains, the trained algorithm was able to detect cotton balls in *in vivo* human studies and other medical foreign objects placed in an *ex vivo* setting. This algorithm has demonstrated its importance in human surgery by locating a cotton ball that was then removed from a patient, not having been known to exist prior to imaging as it was visually indistinguishable from surrounding brain tissue.

**Figure 5:**
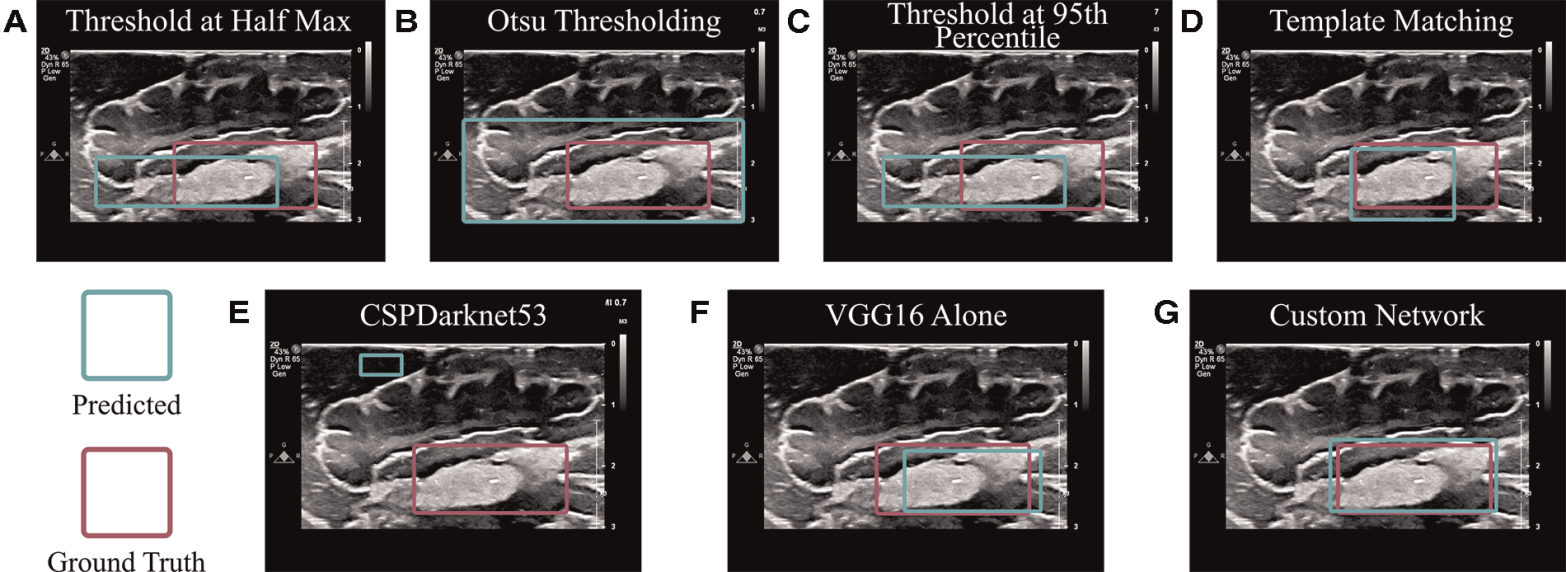
Algorithm comparison. (**A**– **D**) Non-deep learning algorithms were implemented to predict the location of a cotton ball in a neurosurgical ultrasound image. These included (**A**) Thresholding at half the maximum pixel intensity value; (**B**) Otsu thresholding; (**C**) Thresholding at the 95th percentile of the average pixel value within the ground truth bounding box of a training set; and (**D**) Matching the input image to a template image of a cotton ball. (**E**) CSPDarknet53 is the backbone of the YOLOv4 algorithm, which is known for object detection and classification. (**F**) VGG16 is often employed for object localization and was implemented as in (29). (**G**) A custom network was employed using a VGG16 backbone and additional dense network as described.

The acquired dataset of *ex vivo* porcine brain ultrasound images was large and diverse, both of which are necessary qualities for a successful deep learning model. In total, 7,121 images were collected from 10 porcine brains (see Table 1 for a more detailed breakdown). Thresholding and template matching methods that were implemented as control algorithms to verify the necessity for DL were performed both with and without images where a cotton ball was present (i.e., true negatives were either included or excluded). These non-DL methods would likely always report a cotton ball existing, so true negatives were excluded to ensure comparison to the best possible results of thresholding and template matching. However, results including and excluding true negatives for these non-DL methods are both shown for robustness. Specificity is unable to be calculated in cases where true negatives do not exist. Results of each algorithm are displayed in Table 2 and Figure 5. Given that an accurate result is defined here as one with an IoU greater than 50% (25), no control algorithm reached a mean IoU that could be considered accurate without the use of DL. The neural network backbone commonly used in YOLOv4 implementations, CSPDarknet53, surpassed this threshold by 2% using stratified 5-fold CV. The standard VGG16 network without our customization also resulted in a mean IoU of 0.52 using CV.

**Table 1:** Number of images acquired of each size cotton ball for training, validation, and testing of the model.

Cotton ball diameter (mm)	No. of images
0 mm	1,456
1 mm	986
2 mm	878
3 mm	622
5 mm	773
10 mm	820
15 mm	825
20 mm	641
**Total**	**7,121**
Training	4,898
Validation	1,046
Testing	1,057

**Table 2:** Results of Algorithms Tested.

Algorithm	TN images?	Mean IoU	Sensitivity	Specificity	Accuracy
Threshold at half maximum	Yes	0.17	0.21	0.0	0.16
Threshold at half maximum	No	0.41	0.39	—	0.39
Otsu Thresholding	Yes	0.11	0.06	0.0	0.04
Otsu Thresholding	No	0.21	0.09	—	0.10
Threshold at 95th percentile	Yes	0.18	0.24	0.0	0.19
Threshold at 95th percentile	No	0.46	0.48	—	0.49
Template matching	Yes	0.07	0.003	0.0	0.003
Template matching	No	0.19	0.008	—	0.008
CSPDarknet53 (YOLOv4 backbone)	Yes	0.50	0.56	0.0	0.46
5-fold CV of CSPDarknet53	Yes	0.52	0.77	0.0	0.62
VGG16 alone	Yes	0.89	0.99	0.98	0.99
5-fold CV of VGG16	Yes	0.52	0.40	0.60	0.45
Custom network	Yes	0.92	0.99	0.99	0.99
5-fold CV of custom network	Yes	0.94	1.0	1.0	1.0

Control algorithms included thresholding and template matching methods. The threshold at the 95th percentile was calculated using the bounding boxes of ground truth images in the training set. These control algorithms were either performed with or without true negative (TN) images, i.e., images known not to contain a cotton ball. The YOLOv4 backbone, CSPDarknet53, was implemented for comparison as well. Our custom network is a VGG16 backbone with an additional Dense network. Each neural network was additionally implemented and evaluated using stratified 5-fold cross validation (CV). The final custom algorithm achieved a mean IoU of 0.92, or 0.94 after CV.

**Figure 6:**
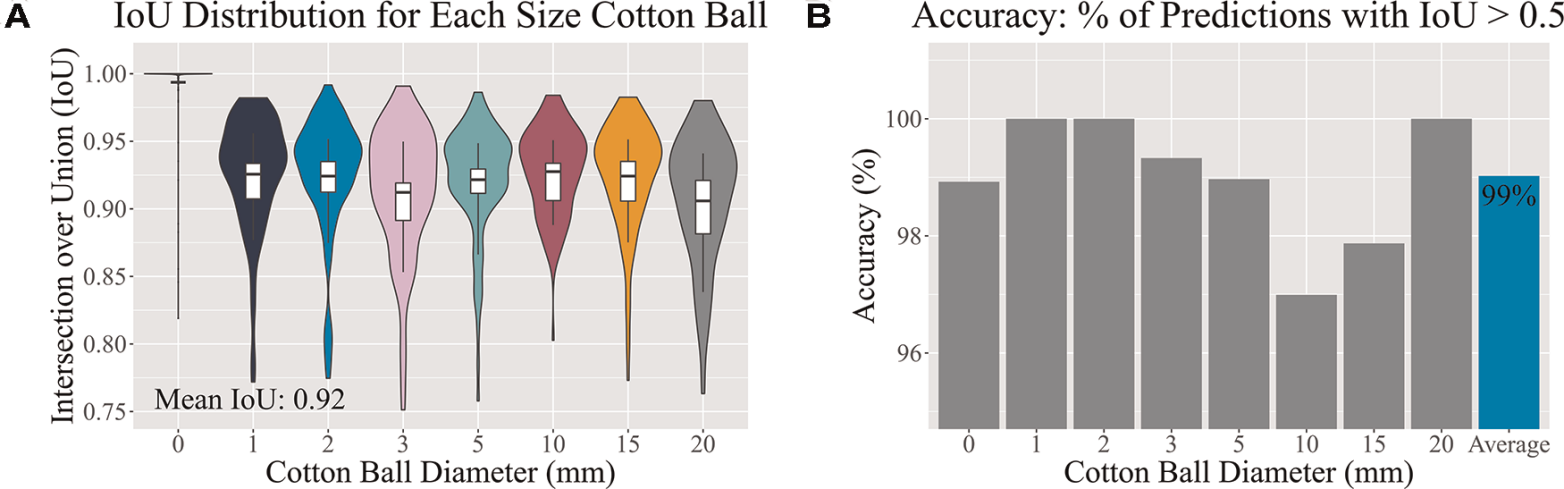
Accuracy assessment. (**A**) A distribution of Intersection over Union (IoU) values of the test set is shown for each implanted cotton ball size. Vertical boxplot lines indicate the 10th and 90th percentiles, while the boxplot itself indicates the 25th, 50th, and 75th percentiles. (**B**) Accurate predictions have IoU values greater than 50%. Here the percentage of accurately predicted bounding boxes are split by cotton ball size.

**Figure 7:**
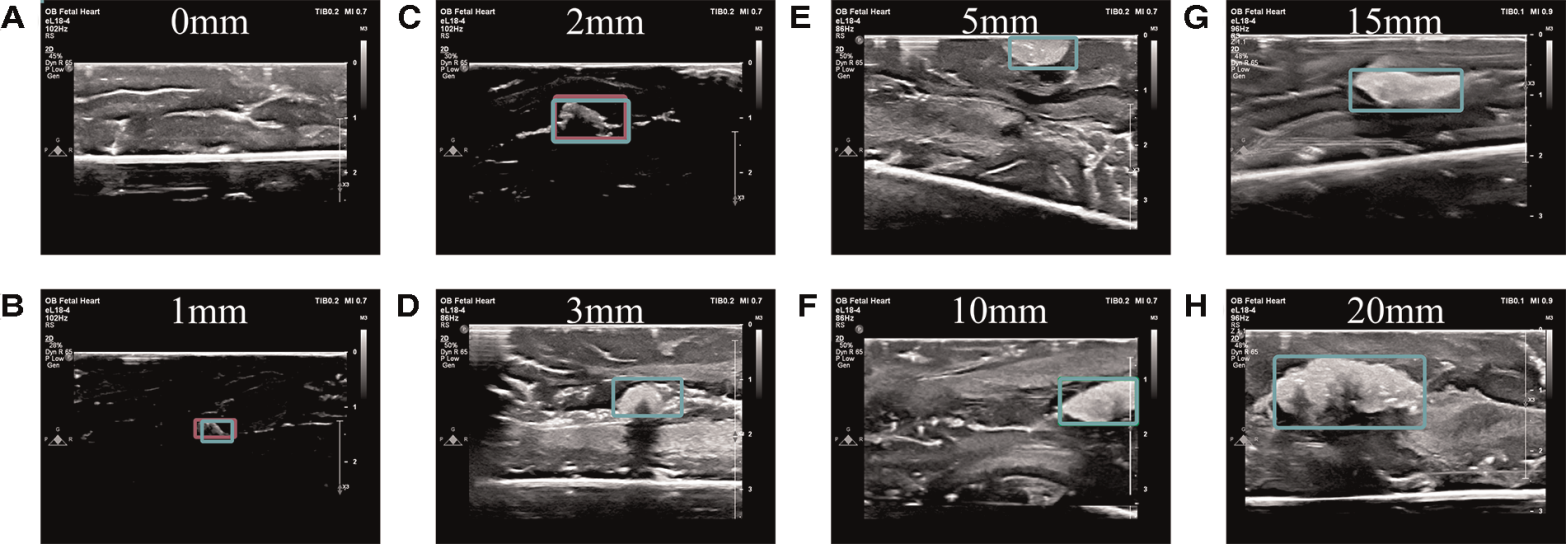
Example predictions. (**A**) No cotton ball is present in this image, nor is one predicted to be present. Predictions of implanted cotton balls with diameter sizes of (**B**) 1 mm, (**C**) 2 mm, (**D**) 3 mm, (**E**) 5 mm, (**F**) 10 mm, (**G**) 15 mm, and (**H**) 20 mm in a fresh porcine brain model are shown by bounding boxes.

Ultimately, the tailored network using a VGG16 backbone and custom dense network described above reached both sensitivity and specificity values of 99% on a hold-out test set. It also resulted in a median IoU of 94%±0.09 and mean IoU of 92% on this test set (Figure 6A). Importantly, the algorithm was 99% accurate (Figure 6B) and correctly identified 92% of the true negative images as not containing a retained foreign body object. Both the training and validation losses (mean squared error) were low at 0.00087 and 0.0018, respectively. When the training and validation losses are similar to each other and low values, the algorithm performs well on all images, whether or not it has “seen” the image before (30). Example predictions of bounding boxes on the ultrasound images are shown in Figure 7.

Stratified 5-fold cross validation of this model reported higher average results than the single reported model. As shown in Table 2, the mean IoU was 0.94 (from 0.93, 0.93, 0.94, 0.95, and 0.95, which were the separate models’ means), while each of the sensitivity, specificity, and accuracy rounded from four significant figures to 100%.

**Figure 8:**
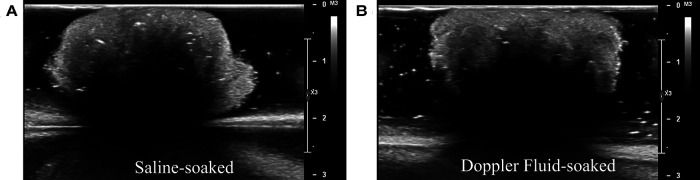
Acoustic comparison of cotton balls. (**A**) Experiments in this study were performed with saline-soaked cotton balls. (**B**) To ensure that cotton would not be visualized differently on an ultrasound machine when absorbing blood rather than saline, the saline-soaked cotton balls were compared to Doppler fluid-soaked cotton balls. Doppler fluid mimics the acoustic properties of blood. (**A**, **B**) are visually similar.

Cotton balls soaked in saline were visually similar to those soaked in blood-mimicking Doppler fluid when captured using ultrasound imaging (see Figure 8). A visual comparison was used to avoid assuming which features were identified by the deep learning algorithm, which uses hidden layers to locate foreign objects. It was also noted that there was only a 1.5% difference in average pixel value between the lighter cotton regions on the ultrasound images as displayed in Figure 8. Although the speed of sound through Doppler fluid (1,570 m/s (31), CIRS, Norfolk, VA, USA) is faster than that of saline solution (approximately 1,500 m/s (32)), these fluids are comparable to the speed of sound through brain tissue (1,546 m/s (33)) but importantly are distinctly different when compared to the speed of sound through a cotton thread (3,130 m/s (34)). The high speed of sound through cotton implies that the fluid in which this material is soaked would have little influence on its visualization via ultrasound imaging. Although typically Doppler fluid is used to measure flow, the comparison between the acoustic properties of blood and Doppler fluid also indicates that these fluids are similar when stagnant as well, which would be the case during a surgery. Blood and Doppler fluid have similar speeds of sound (1,583 and 1,570 m/s, respectively), densities (1,053 and 1,050 kg/m${}^{3}$, respectively), attenuation coefficients (0.15 and 0.10 dB/(cm MHz), respectively), viscosities (3 and 4 mPa⋅s, respectively), particle sizes (7 and 5 mm, respectively), and backscatter coefficients (0 and $10^{-30}$, respectively) (31,35,36). They differ primarily in that blood is non-Newtonian whereas Doppler fluid is Newtonian, though this characteristic does not affect intraoperative ultrasound imaging when the blood is stagnant in the cranial cavity (36). As a result, it is understood that the echo generated by still Doppler fluid would accurately represent an echo generated by blood.

**Figure 9:**
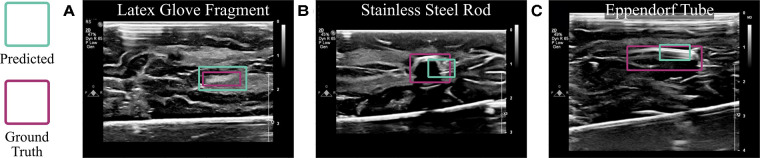
Algorithmic implementation using other materials. The trained model, without any changes, was used to detect (**A**) latex glove fragments, (**B**) a stainless steel rod, and (**C**) an Eppendorf tube implanted into the brain.

The algorithm, without any changes or additional training, was also able to detect other objects placed in or around the brain. As shown in Figure 9, it localized a fragment of a latex glove with an IoU of 0.88, a short rod of stainless steel with an IoU of 0.68, and an Eppendorf tube with an IoU of 0.40. Although these objects were visually distinguishable from the brain tissue unlike cotton balls, this experiment proves that the ultrasound-based technology described here is beneficial in numerous use cases.

**Figure 10:**
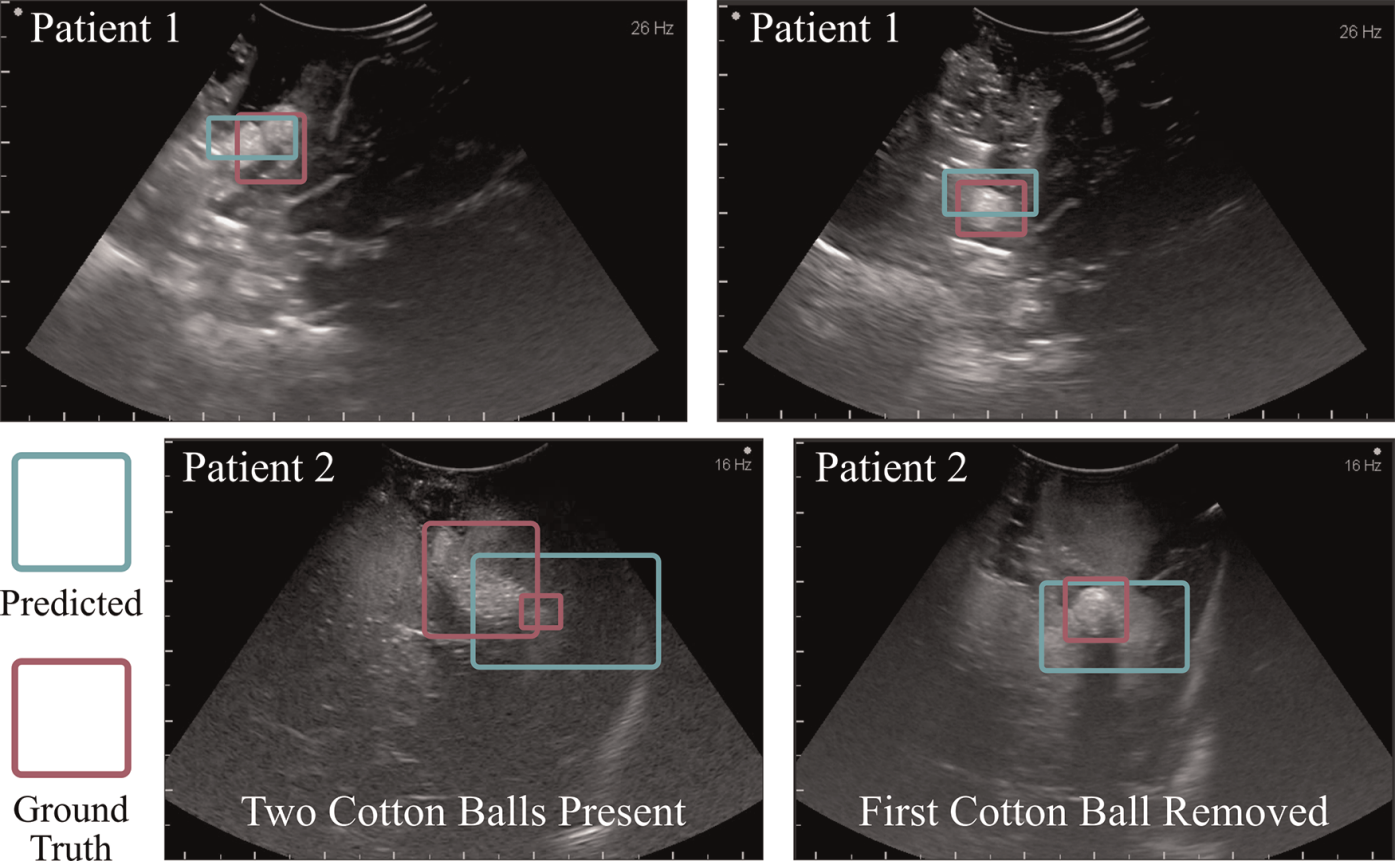
Algorithmic implementation in humans. The trained model, without any changes beyond initial contrast enhancement of the images, was used to detect cotton balls in a human brain following neurosurgery. The top two example images were taken towards the end of an aneurysm surgery. The bottom two images were captured following a tumor resection procedure. One cotton ball was known to be placed within the cavity (left), and upon removal of this object a second cotton ball was found (right). This study prevented accidental retention of an unidentified foreign body object. Intentionally placed cotton balls had a diameter of 10 mm prior to placement within the cavity, which alters the shape. The initially unseen cotton ball in Patient 2 was 5 mm in diameter.

Importantly, the algorithm demonstrated the ability to prevent accidental foreign body retention and to detect cotton balls in ultrasound images captured during human neurosurgical procedures. The cotton balls placed deliberately for visualization via ultrasound during the cases (one per case) were accurately identified (see Figure 10). During the second case (Patient 2), when intending to capture a true negative image, an initially unidentified foreign body object was able to be seen in the operation site. This final ultrasound scan informed the neurosurgeons that they should explore the cavity once again. Following an extensive search, a small cotton ball approximately 5 mm in diameter was located underneath a gyral fold. This patient, undergoing a second brain surgery already this year, was protected from a third surgery that could have resulted from a retained cotton ball. This algorithm was tested post-operatively on the images captured during this surgery and accurately located both cotton balls (Figure 10). From left to right, the IoUs of the example images for Patient 1 in Figure 10 were 0.86 and 0.91. The IoUs of Patient 2’s image with two cotton balls present were 0.72 for the larger cotton ball and 0.69 for the smaller, hidden cotton ball. The image on the right-hand side displays only this smaller, once hidden cotton ball, and the algorithm predicted its presence with an IoU of 0.83. The algorithm was unable to identify true negative images for the human studies. However, the Aloka UST-9120 probe used to capture these images has an operating frequency of 7MHz, compared to the Philips eL18-4 operating frequency of 11MHz. Decreased frequency corresponds to lower resolution, thus indicating an approximately 50% loss in image quality of the human study compared to the *ex vivo* study.

The algorithm was implemented into intuitive web and smartphone applications. A clinician may upload an image to either application, after which the application runs the trained algorithm in the back-end. In 0.38 s, the web application is able to predict, localize, and display bounding boxes on the captured ultrasound images (see Supplementary Video Figure S11). The smartphone application offers the additional feature of being able to capture an image of the screen of an ultrasound machine and immediately check this image for cotton balls, running in approximately 1 s (see Supplementary Video Figure S12).

## Discussion

The ultrasound-based technology presented here identifies cotton balls in the absence of injections, dyes, or radiofrequency tags and is based on clinical workflow. Cotton balls, a common item used in the operating room, serve as a model for foreign body objects that may lead to severe immunologic responses if retained post-surgery. Overcoming the visual barriers of distinguishing blood-soaked cotton from brain tissue, ultrasound imaging captured what other modalities could not: the contrasting acoustic properties of cotton in relation to brain tissue. Using thousands of acquired *ex vivo* porcine brain images demonstrating this contrast, a deep neural network learned the unique features of cotton in an ultrasound image and successfully output bounding boxes to localize the foreign bodies with a median IoU of 0.94±0.09 and 99% accuracy. This algorithm automated the translation of over 700,000 data points (the number of pixels in each image prior to preprocessing) to four simple numbers describing the location and size of a retained surgical item in the brain. Because gossypibomas may result from fragments of cotton (37), the work here takes caution in localizing pieces of cotton down to 1mm in diameter. The potentially life-saving capability of this study was exhibited explicitly during the second in-human data collection. The neurosurgeons had placed a cotton ball, taken an ultrasound scan, and subsequently removed it, yet there remained an unidentified foreign body object clearly visible in the image. Upon searching, they located a cotton ball that had been tucked behind a gyral fold and not initially seen by the surgeon. This object was found because they elected to perform an intraoperative ultrasound. In the future, implementing the algorithm developed here will ensure rapid and confident diagnosis of a retained foreign object.

There has only been one previous report of an algorithm for the automatic detection of foreign body objects (15). However, the dataset acquired in Mahapatra et al. (15) was unrepresentative of a clinical setting and showed minimal variation between images, which risks overfitting. In contrast, the work described here captured all images in a manner more conducive to deep learning: sizes and locations of implanted cotton in the brain were all varied, and deformation of cotton as it absorbed saline additionally added shape variability to the images. Another benefit of this work is that all *ex vivo* images were acquired in a rubber-lined container to attenuate noise and avoid artifacts. Additionally, this technology is intended for clinical implementation; therefore an ultrasound machine readily available and approved for hospital use, a Philips EPIQ 7, was used. Further, this algorithm accurately localizes any size cotton ball without the added computational expense of labeling cotton size as in YOLOv4, which was used in Mahapatra et al. (15), since this label is redundant in medical images with known scales. To show that the custom neural network described here improved upon Mahapatra et al. (15), the backbone of YOLOv4 (i.e., CSPDarknet53) was trained and tested on the newly acquired image dataset. YOLOv4 is typically implemented to identify multiple different types (or classes) of objects in an image, and therefore is computationally expensive in comparison to our smaller, custom network. CSPDarknet53 is specific to localization rather than classification. Therefore, because the specific task here is to localize cotton balls rather than distinguish or classify different objects within the cranium, we did not re-implement the additional layers (known as the neck and head) of YOLOv4. CSPDarknet53 was approximately half as accurate as our custom network. Our study also demonstrated the first working example of automated foreign body object detection in humans.

There are a few limitations to this work that serve as future steps in establishing this technology in the clinic. Currently, the algorithm will identify only one cotton ball per image. If there are two, for example, it will identify one of them, and upon its extraction out of the brain, identify the other. Clumped cotton balls also appear to the neural network as one singular, larger object as demonstrated in Figure 10 Patient 2; though importantly, it recognized the presence of a foreign body. Future work should allow for multi-object detection. In addition, a few modifications can make for improved clinical translation following the first two successful implementations in humans reported here. For example, a database of ultrasound images with cotton balls used during human neurosurgery should be acquired and tested with a fine-tuned version of the algorithm presented. This database should incorporate all of the brands of clinical machines and types of probes one might find in a neurosurgical setting. The somewhat low accuracy of *in vivo* data compared to *ex vivo* results is likely due to the decreased image quality, which was cut in half *in vivo* due to unavailability of a more modern system or higher frequency probe, and due to the use of a curvilinear probe as opposed to linear, which the algorithm had never seen in training.

Foreign body objects could be localized using this algorithm regardless of the anatomical region, for example in abdominal, vascular, or orthopedic procedures, etc. (38–43). Beyond cotton balls, ceramic, silicone, metal, or hydrogel implants may trigger foreign body responses that demand prompt care (44,45). One of the first steps in treatment would be localization of the foreign body object, which could be accomplished with this technology. In this study, the *ex vivo* data collected demonstrated the same accuracy, sensitivity, and specificity whether or not images were filtered in pre-processing, though the methods used show promise in increasing accuracy when blurrier or poorer quality images were captured such as the *in vivo* data. As was demonstrated by the detection of other foreign bodies and success in humans, this algorithm is flexible as trained, and its applications could be expanded using simple fine-tuning methods. Anatomical modifications that may have occurred during surgery, which one might imagine could impact clinical translation, did not cause a noticeable issue. This algorithm searches for cotton rather than patterns in brain tissue, and neurosurgeons are unlikely to considerably change the gyral folds that may be present. Additionally, the neurosurgeon added saline to the cranial cavity, thereby removing any potential air gaps that could distort the images *in vivo*. Similarly, pooled blood resulting from the surgery did not and would not effect the ultrasound images because it has a similar speed of sound as saline or water, meaning that it is anechoic or hypoechoic whereas cotton is hyperechoic. Therefore, the blood would serve to further distinguish the cotton from the surrounding anatomy. Following the scanning protocol presented ensures the entire region of interest will be covered. This work could additionally benefit ultrasound uses in industry such as nondestructive testing (46,47).

## Conclusions

Ultrasound is an inexpensive, non-ionizing, and well-established imaging modality across medical fields. It provides insight into the acoustic properties of different structures in the body, including foreign objects left behind during brain surgery. This work described a rapid and accurate technology that uses ultrasound imaging and is capable of localizing such foreign objects intraoperatively in humans. The importance of this work is emphasized by the fact that a cotton ball not seen by the neurosurgeon during a human procedure was located as a result of conducting ultrasound imaging for this study, thereby preventing immunologic reactions in the patient, expensive follow-up surgery, and a potential malpractice lawsuit.

## Data Availability

The raw data supporting the conclusions of this article will be made available by the authors, without undue reservation.
